# Supplementary material for: Hybracter: enabling scalable, automated, complete and accurate bacterial genome assemblies
Source: Microb Genom. 2024 May 8;10(5):001244. doi: 10.1099/mgen.0.001244 (PMC11165638; doi:10.1099/mgen.0.001244)

Figure 1: Outline of the Hybracter workflow.

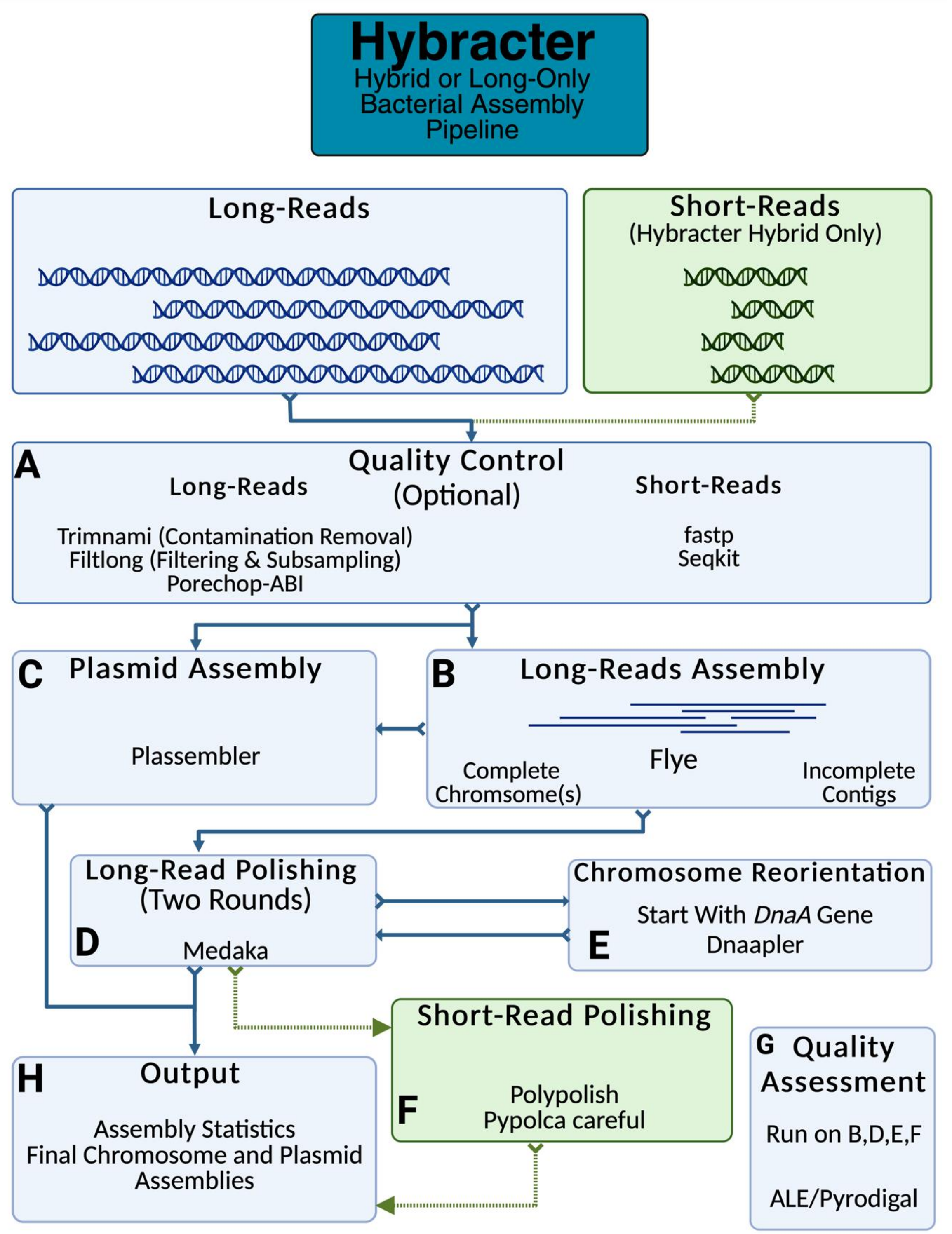

Figure 2: Comparison of the counts of single nucleotide variants (SNVs) and small (<60bp) insertions and deletions (InDels) (A) and the total number of large (>60bp) InDels (B) for the hybrid tools benchmarked (Hybracter hybrid in dark blue, Dragonflye hybrid in orange and Unicycler in green). The counts of SNVs and small InDels (C) and the total number of large InDels (D) for the long tools benchmarked (Hybracter long in light blue, Dragonflye long in grey) are also shown. All data presented is from the benchmarking output run with 8 threads.

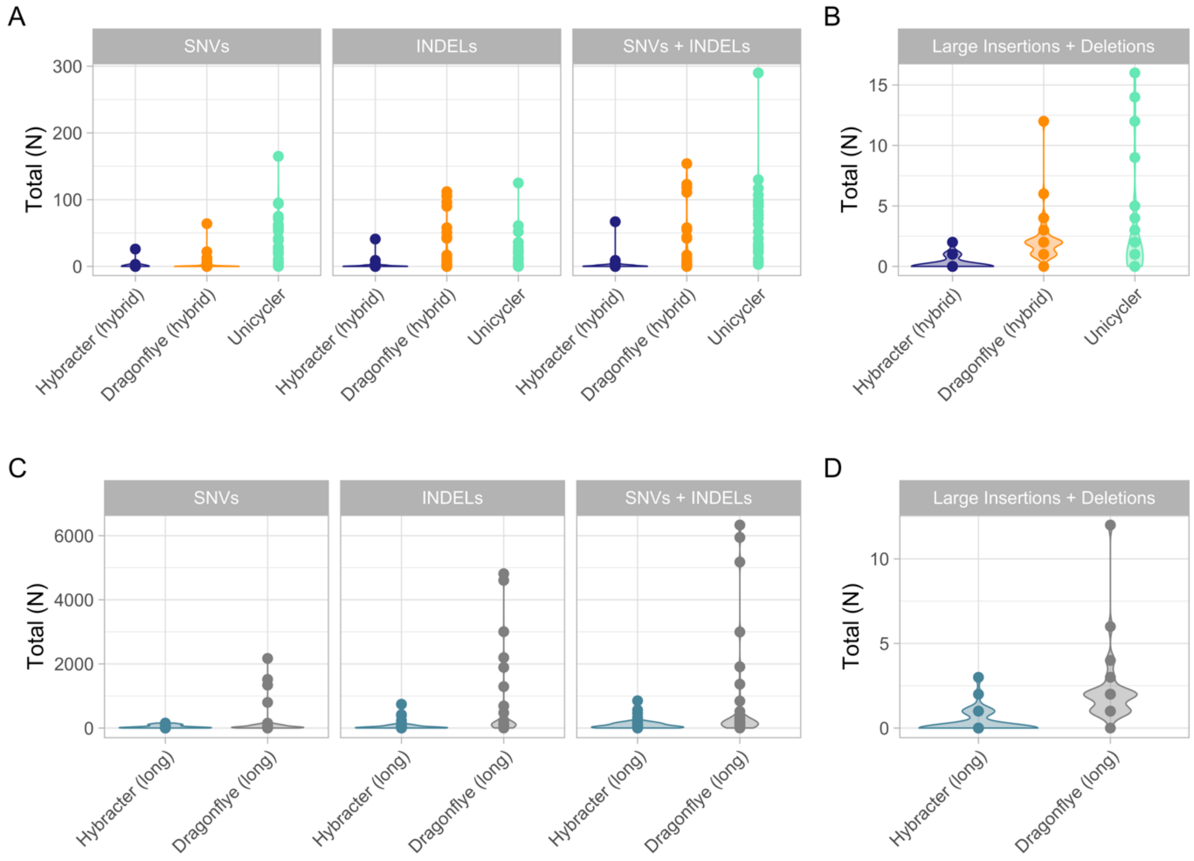

Figure 3: Comparison of wall-clock runtime (in seconds) of Hybracter hybrid, Dragonflye hybrid, Unicycler, Hybracter long and Dragonflye long when run with 8 and 16 threads.

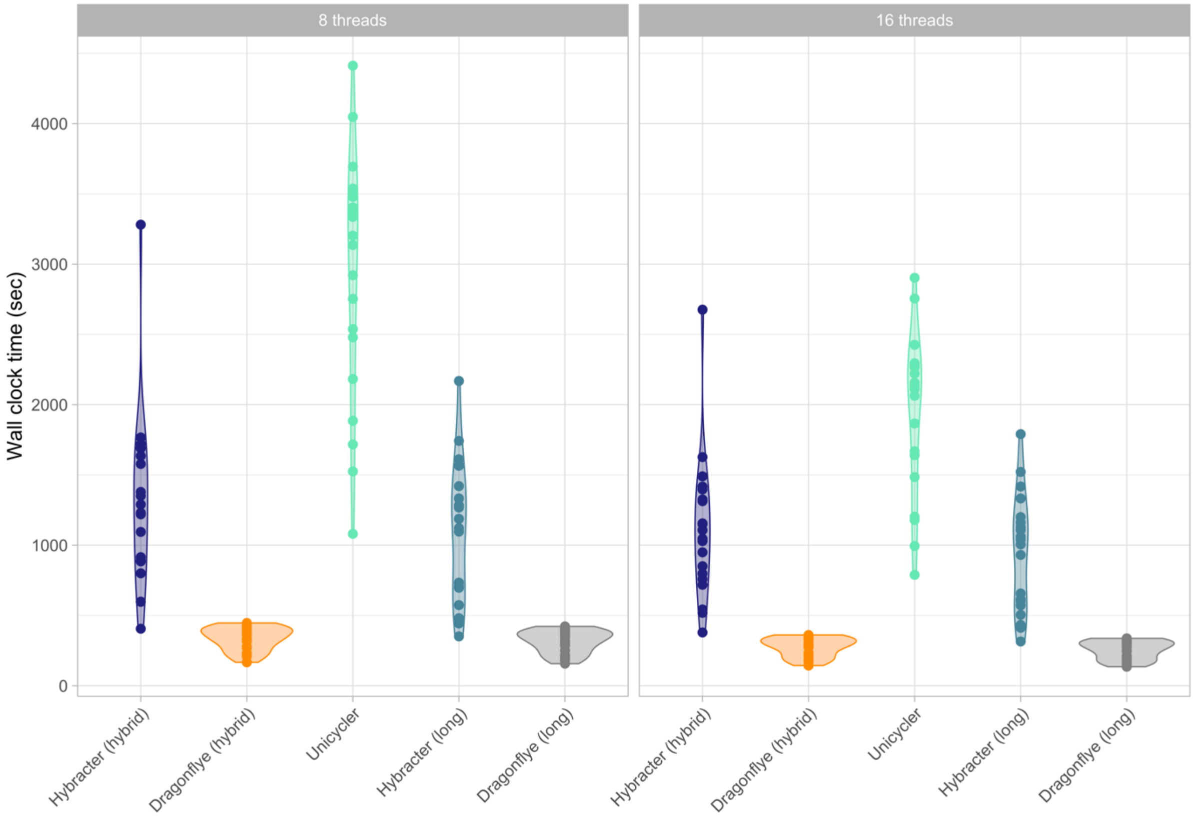

Figure 4: Comparison of the counts of small (<60bp) (A) and large (>60bp) (B) insertions and deletions (InDels) and SNVs (C) for Hybracter hybrid, Dragonflye hybrid, Unicycler, Hybracter long and Dragonflye long chromosome assemblies of *Lerminiaux* Isolate B (*Enterobacter cloacae*) at 5x intervals of sequencing depth from 10x to 100x.

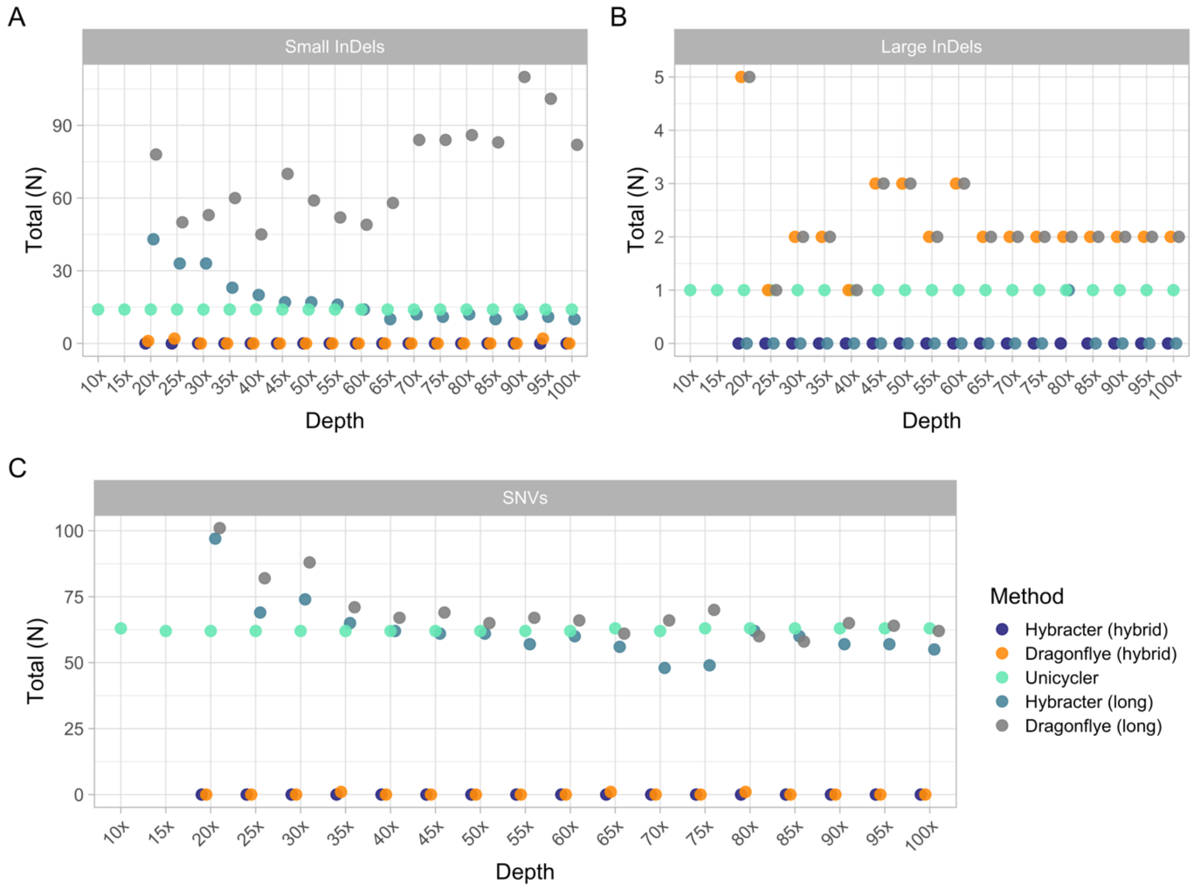

Supplementary Figure 1: For lower quality samples only (all five ATCC fast model base-called samples, *S. aureus* JKD6159 with R9 chemistry and *M.* tuberculosis H37R2), this figure presents a comparison of the counts of small nucleotide variants (SNVs) and small (<60bp) insertions and deletions (InDels) (A) and the total number of large (>60bp) InDels (B) for the hybrid tools benchmarked (Hybracter hybrid in dark blue, Dragonflye hybrid in orange and Unicycler in green). The counts of SNVs and small InDels (C) and the total number of large InDels (D) for the long tools benchmarked (Hybracter long in light blue, Dragonflye long in grey) are also shown. All data presented is from the benchmarking output run with 8 threads.

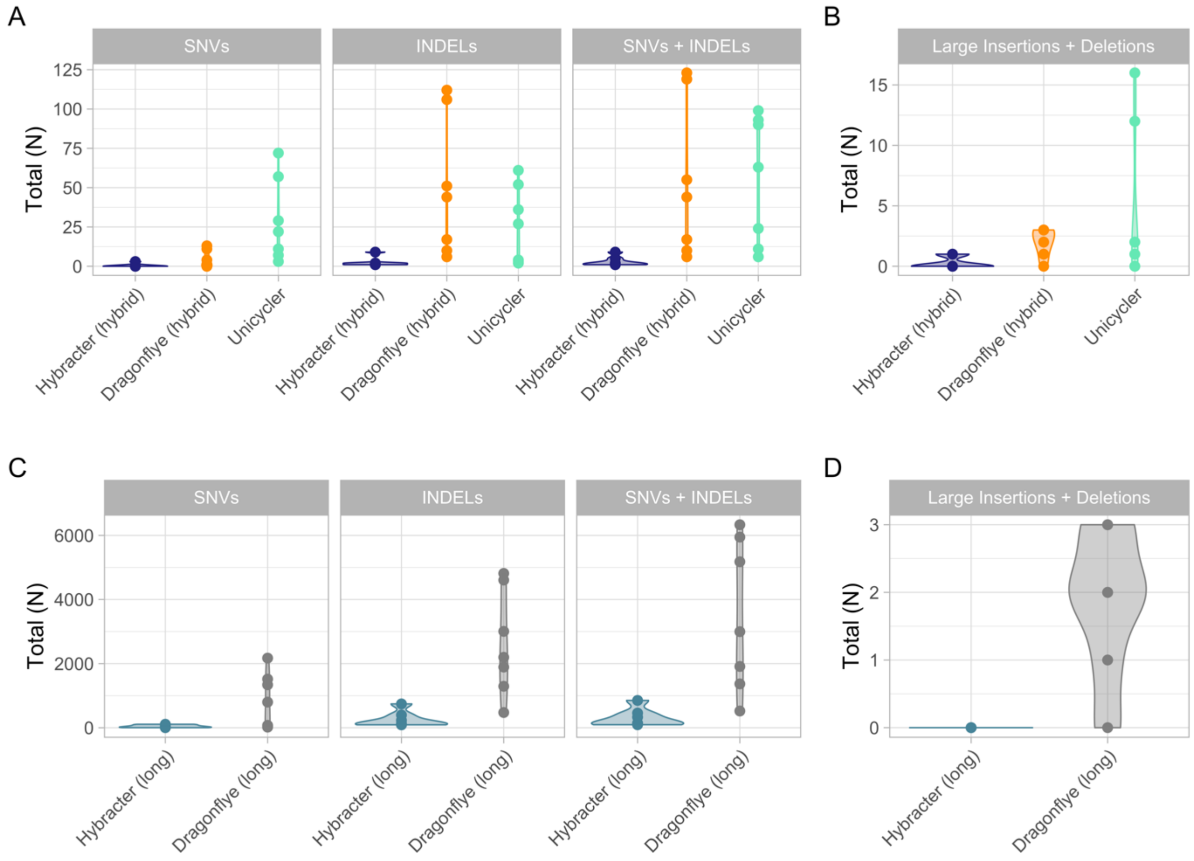

Supplementary Figure 2: For simplex super-accuracy model basecalled samples (five ATCC, *S. aureus* JKD6159 with R10 chemistry and all twelve *Lerminiaux* et al. samples), this figure presents a comparison of the counts of small nucleotide variants (SNVs) and small (<60bp) insertions and deletions (InDels) (A) and the total number of large (>60bp) InDels (B) for the hybrid tools benchmarked (Hybracter hybrid in dark blue, Dragonflye hybrid in orange and Unicycler in green). The counts of SNVs and small InDels (C) and the total number of large InDels (D) for the long tools benchmarked (Hybracter long in light blue, Dragonflye long in grey) are also shown. All data presented is from the benchmarking output run with 8 threads.

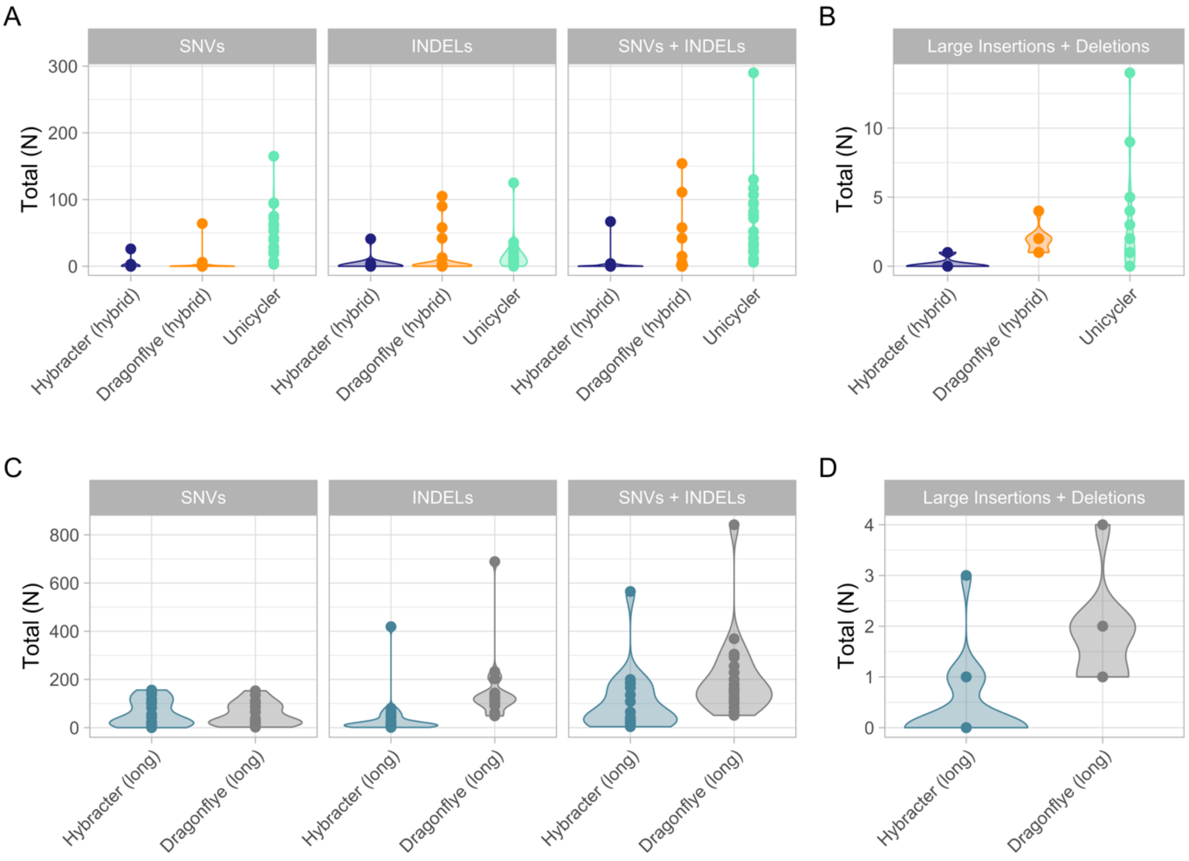

Supplementary Figure 3: For duplex super-accuracy model basecalled samples (five ATCC samples), this figure presents a comparison of the counts of small nucleotide variants (SNVs) and small (<60bp) insertions and deletions (InDels) (A) and the total number of large (>60bp) InDels (B) for the hybrid tools benchmarked (Hybracter hybrid in dark blue, Dragonflye hybrid in orange and Unicycler in green). The counts of SNVs and small InDels (C) and the total number of large InDels (D) for the long tools benchmarked (Hybracter long in light blue, Dragonflye long in grey) are also shown. All data presented is from the benchmarking output run with 8 threads.

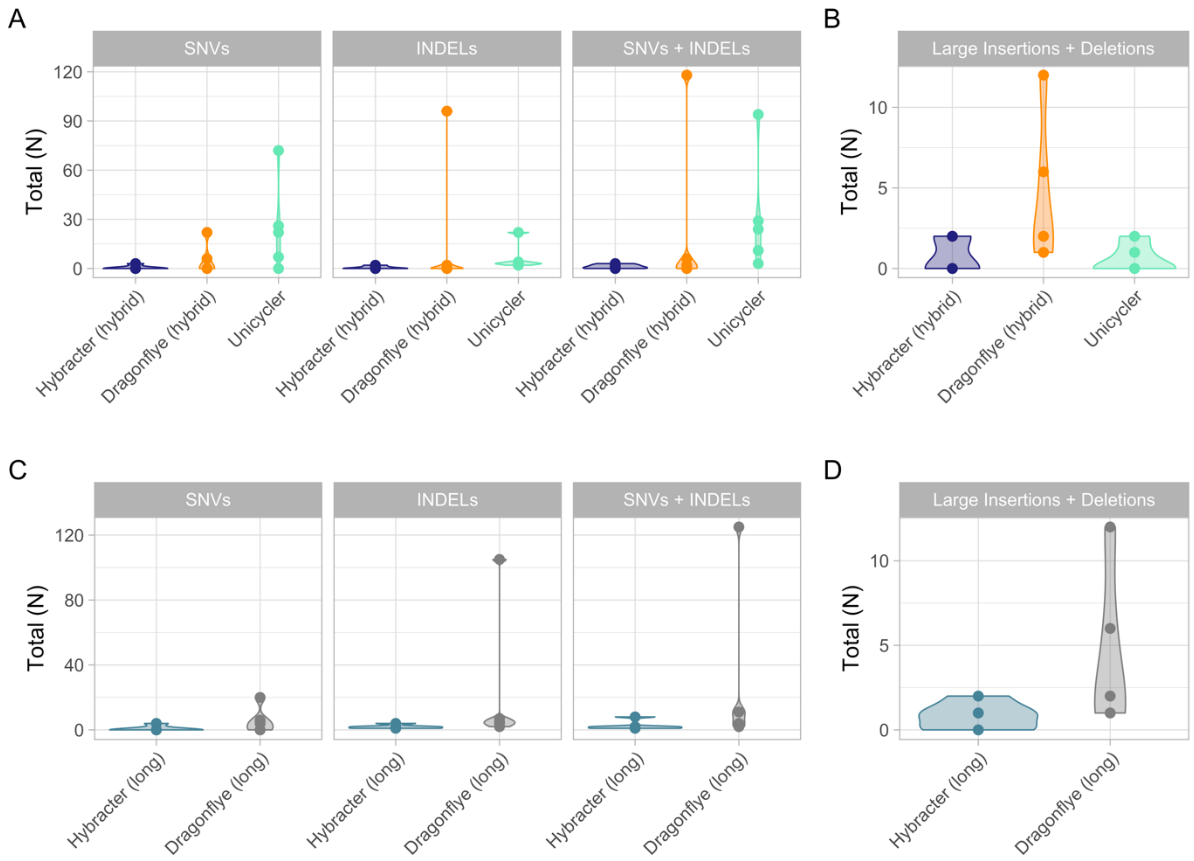

Supplement: Uncited Fig. S1. [file mgen-10-01244-s002.pdf]
